# A New Estimation of Global Soil Greenhouse Gas Fluxes Using a Simple Data-Oriented Model

**DOI:** 10.1371/journal.pone.0041962

**Published:** 2012-08-02

**Authors:** Shoji Hashimoto

**Affiliations:** Department of Forest Site Environment, Forestry and Forest Products Research Institute (FFPRI), 1 Matsunosato, Tsukuba, Ibaraki, Japan; University of Illinois, United States of America

## Abstract

Soil greenhouse gas fluxes (particularly CO_2_, CH_4_, and N_2_O) play important roles in climate change. However, despite the importance of these soil greenhouse gases, the number of reports on global soil greenhouse gas fluxes is limited. Here, new estimates are presented for global soil CO_2_ emission (total soil respiration), CH_4_ uptake, and N_2_O emission fluxes, using a simple data-oriented model. The estimated global fluxes for CO_2_ emission, CH_4_ uptake, and N_2_O emission were 78 Pg C yr^−1^ (Monte Carlo 95% confidence interval, 64–95 Pg C yr^−1^), 18 Tg C yr^−1^ (11–23 Tg C yr^−1^), and 4.4 Tg N yr^−1^ (1.4–11.1 Tg N yr^−1^), respectively. Tropical regions were the largest contributor of all of the gases, particularly the CO_2_ and N_2_O fluxes. The soil CO_2_ and N_2_O fluxes had more pronounced seasonal patterns than the soil CH_4_ flux. The collected estimates, including both the previous and the present estimates, demonstrate that the means of the best estimates from each study were 79 Pg C yr^−1^ (291 Pg CO_2_ yr^−1^; coefficient of variation, CV = 13%, *N* = 6) for CO_2_, 21 Tg C yr^−1^ (29 Tg CH_4_ yr^−1^; CV = 24%, *N* = 24) for CH_4_, and 7.8 Tg N yr^−1^ (12.2 Tg N_2_O yr^−1^; CV = 38%, *N* = 11) for N_2_O. For N_2_O, the mean of the estimates that was calculated by excluding the earliest two estimates was 6.6 Tg N yr^−1^ (10.4 Tg N_2_O yr^−1^; CV = 22%, *N* = 9). The reported estimates vary and have large degrees of uncertainty but their overall magnitudes are in general agreement. To further minimize the uncertainty of soil greenhouse gas flux estimates, it is necessary to build global databases and identify key processes in describing global soil greenhouse gas fluxes.

## Introduction

Soil greenhouse gas (GHG; particularly CO_2_, CH_4_, and N_2_O) fluxes are a key component to understanding climate change. CO_2_ is produced by mostly heterotrophic organisms and plant root respiration and is emitted from the soil surface to the atmosphere [1]–[2]. Soil is generally a sink of atmospheric CH_4_ through oxidation in the soil [3]–[4], but the soil in wetlands is a strong source of CH_4_. In general, N_2_O is released from the soil surface to the atmosphere [5]–[6] and is the result of N_2_O production and consumption processes in soil [7]. The soil CO_2_ flux is the largest component of the soil GHG fluxes, and it nearly counterbalances the plant carbon fixation. However, considering their global warming potentials, CH_4_ and N_2_O fluxes are also important components. Moreover, it is reported that recent changes in the climate may increase these soil GHG fluxes both globally and regionally [2] [8].

Despite the importance of these soil GHG fluxes, the number of reports on global soil GHG fluxes remains limited. In general, these estimations have been performed using detailed process-oriented models [6] [9] or simple data-oriented models [2] that entail data synthesis, and these two approaches compensate for the disadvantages of each. For example, simple data-oriented models cannot trace detailed processes and may not be suitable for long-term predictions, but they can provide more data-oriented estimates. Also, simple data-oriented models provide benchmarks against results from more detailed, process-oriented models [1] [10].

The objective of this paper is to report new global estimates of soil CO_2_ emission (total soil respiration), CH_4_ uptake, and N_2_O emission fluxes. First, I report new global estimates that were estimated using a simple data-oriented model [8] [11]. The soil GHG flux submodels describe each gas flux simply in terms of three functions: the soil physiochemical properties, water-filled pore space, and soil temperature. The total fluxes, spatial distribution, and seasonality of each flux were estimated. Here, the average fluxes between 1980 and 2009 are provided. Second, the global estimates reported in previous studies were compiled, and I report the means of the best estimates from each study.

## Results

The estimated global fluxes of CO_2_ emission, CH_4_ uptake, and N_2_O emission were 78 Pg C yr^−1^ (Monte Carlo 95% confidence interval, 64–95 Pg C yr^−1^), 18 Tg C yr^−1^ (11–23 Tg C yr^−1^), and 4.4 Tg N yr^−1^ (1.4–11.1 Tg N yr^−1^), respectively. The uncertainty was the largest for the N_2_O flux and smallest for the CO_2_ flux. Respectively, the boreal (mean annual temperature, T<2.0°C), temperate (2.0≤T≤17.0°C), and tropical (T>17.0°C) ecosystems contribute 10%, 19%, and 70% to the total global CO_2_ flux, 18%, 26%, and 56% to the total global CH_4_ flux, and 5%, 18%, and 77% to the total global N_2_O flux. The contribution of the tropical ecosystems was the highest for all of the gases, especially for CO_2_ and N_2_O.

**Figure 1:**
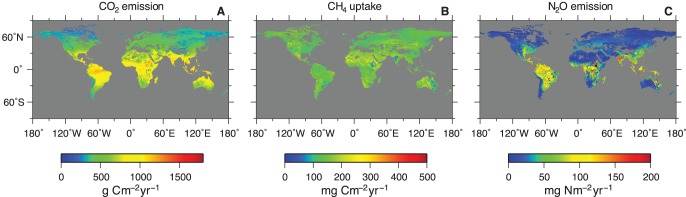
Global maps of the estimated rates of fluxes. (**A**) CO_2_ emission flux, (**B**) CH_4_ uptake flux, and (**C**) N_2_O emission flux. The values are the averages between 1980 and 2009.

**Figure 2:**
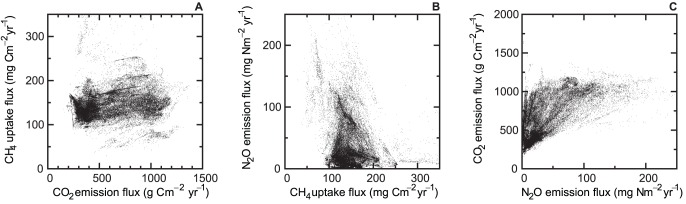
Relationships between each flux. (**A**) CO_2_ emission flux and CH_4_ uptake flux, (**B**) CH_4_ uptake flux and N_2_O emission flux, and (**C**) N_2_O emission flux and CO_2_ emission flux.

**Figure 3:**
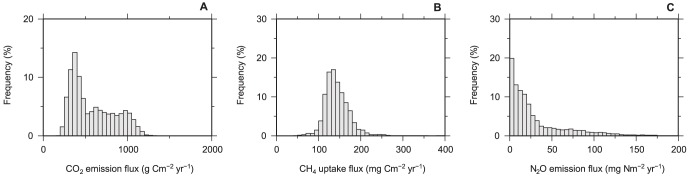
Histograms of modeled soil GHG fluxes by gridded cells. (**A**) CO_2_ emission flux, (**B**) CH_4_ uptake flux, and (**C**) N_2_O emission flux.

Figure 1 shows the estimated spatial distributions of the soil CO_2_ emission, CH_4_ uptake, and N_2_O emission fluxes; the relationships between each gas flux are shown in Figure 2. The CO_2_ and N_2_O fluxes showed clear spatial patterns that were controlled mainly by temperature. The fluxes were higher in the tropical regions, and they decreased at higher latitudes, yet the two gas fluxes do not always co-occur (Figure 1AC and Figure 2C). The fluxes from the +30° to −30° latitude belt were high for CO_2_ and N_2_O, but the belt seems to be wider for CO_2_ than N_2_O. For N_2_O at the latitude regions of approximately +30° and −30°, only the fluxes from east of North America and East Asia, east of South America, and east of Australia were high. In contrast, the CH_4_ flux did not show clear temperature-induced spatial patterns. Hot spots of CH_4_ uptake were observed in North and South America, Kamchatka, Japan, and New Zealand, corresponding to the distribution of highly porous soils (Andosols). The distribution patterns of the frequencies differed among the three gases (Figure 3). The CO_2_ flux showed a wider and flatter range than the CH_4_ flux and exhibited a relatively low peak value (300–450 g C m^−2^ yr^−1^). The CH_4_ flux has a single peak in the middle of the range. The N_2_O flux had a long, right-skewed distribution, which is often observed in field studies [12]. The distributions in the histograms correspond to the spatial distribution of each gas flux. The distinct spatial distribution patterns for CO_2_ and N_2_O (Figure 1) resulted in the broad distributions of the CO_2_ and N_2_O fluxes in the histograms (Figure 3); the wide spatial distribution of the smaller flux resulted in peaks in the low values in CO_2_ and N_2_O flux histograms (Figure 3).

**Figure 4:**
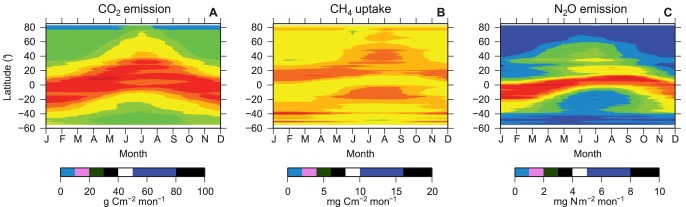
Seasonal and latitudinal distributions of the fluxes. (**A**) CO_2_ emission flux, (**B**) CH_4_ uptake flux, and (**C**) N_2_O emission flux.

Seasonal changes in the CO_2_ emission, CH_4_ uptake, and N_2_O emission fluxes are shown in Figure 4. Except in low-latitude regions, CO_2_ and N_2_O showed clear seasonality, being high during the summer and low during the winter. As observed in Figure 1, the belt of large flux around the tropical regions was narrower for N_2_O than CO_2_, and a north-south asymmetry can be observed for N_2_O. The seasonal changes in the CH_4_ flux were not as large as the other two gases. The CH_4_ uptake flux was relatively higher in the middle latitudes and was high during the summer and low during the winter. The seasonality seemed to be the opposite at low latitudes (+20° and −20°).

## Discussion

**Figure 5:**
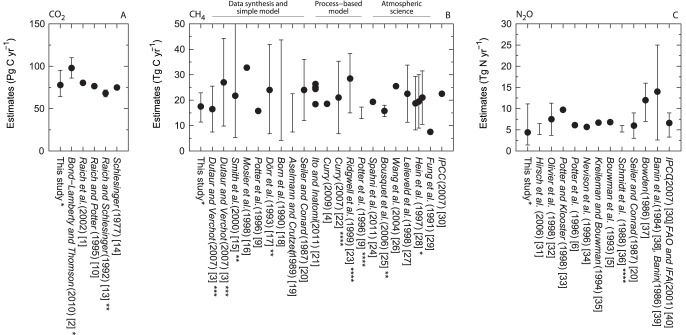
Comparison of the global estimates for each flux. (**A**) CO_2_ emission flux, (**B**) CH_4_ uptake flux, and (**C**) N_2_O emission flux. The estimates are in reverse chronological order. For the CH_4_ flux, the studies were divided according to the methodologies because the number of studies was large. The values in “data synthesis and simple model” include estimates from data synthesis and extrapolations. For the CO_2_ flux, all estimates are from data synthesis and simple modeling. For the N_2_O flux, only the estimate in *Hirsch et al.* (2006) [31] is from atmospheric inversion, and the estimates from *Potter and Klooster* (1998) [33] to *Bouwman et al.* (1993) [5] are from process-based model. Other estimates are from data synthesis. The definitions of the bars differ (*95% confidence interval; **standard deviation; ***standard error; ****based on two different model assumptions or parameters; no-mark: no uncertainty was reported or the definition of the bar could not be explicitly identified.). The higher end of the bar of *Smith et al.* (2000) [15] is 90 Tg C yr^−1^ (**B**). The values in *Ito and Inatomi* (2011) [21] are the results from four models (**B**). The values in *Hein et al.* (1997) [28] are the results from three different assumptions (**B**). The value in *Hirsch et al.* (2006) [31] is the preindustrial flux (i.e., the anthropogenic terrestrial flux enhancement was removed), and the value in *Olivier et al.* (1998) [32] is the sum of the soil microbial production, grasslands, and background emissions arable land sources (**C**). For *Banin et al.* (1984) [38] and *Banin* (1986) [39], the estimate without cultivated land is plotted (**C**). When cultivated land is include, the estimate ranges from 4 to 29 Tg N yr^−1^. For the estimates of IPCC, only the latest estimates were included (*IPCC*, 2007) [30] (**B**,**C**). In this synthesis, I did not include estimates that appeared to be the citation of the estimates in IPCC reports. *Bouwman et al.* (1995) [41] reported two estimates of N_2_O emission flux that were calculated by overlaying the emission inventories from *Bouwman et al.* (1993) [5] and *Kreileman and Bouwman* (1994) [35] with a new land cover database. The estimates (7.0 and 6.6 Tg N yr^−1^) were slightly different from original estimates (6.8 and 6.7 Tg N yr^−1^), but were approximately the same as the originals; therefore, these estimates of *Bouwman et al.* (1995) [41] were not included in this compilation.

I compiled reports on global soil CO_2_ emission, CH_4_ uptake, and N_2_O emission fluxes [1]–[6] [9]–[10] [13]–[41], and the estimates in this study were comparable to those of previous studies (Figure 5). The estimate for CO_2_ was within the range of previous studies but was relatively smaller than the latest estimate derived from the synthesis of global data [2]. For the CH_4_ uptake, the estimate in this study was intermediate among the previous estimates, and the CH_4_ estimates had greater variance when compared with the CO_2_ estimates. In my literature survey, the number of estimates for CH_4_ was the largest among the three gases. The estimate for N_2_O was of the same magnitude as the previous estimate but was relatively smaller than those of previous studies. When evaluating the uncertainty of each study, the uncertainties for the N_2_O and CH_4_ estimates were quite large. The uncertainty for the CO_2_ flux appears to be smallest; however, it should be emphasized that the uncertainty for the CO_2_ estimate would still have the highest impact on the uncertainty in terms of the global GHG budget because, among the three gases, the soil CO_2_ efflux is the largest component in global warming potentials. The means of the best estimates from each study were 79 Pg C yr^−1^ (291 Pg CO_2_ yr^−1^; coefficient of variation, CV = 13%, *N* = 6) for CO_2_, 21 Tg C yr^−1^ (29 Tg CH_4_ yr^−1^; CV = 24%, *N* = 24) for CH_4_, and 7.8 Tg N yr^−1^ (12.2 Tg N_2_O yr^−1^; CV = 38%, *N* = 11) for N_2_O. For N_2_O, the earliest two estimates (the estimate of *Banin et al.* (1984) [38] and *Banin* (1986) [39], and that of *Bowden* (1986) [37]) are markedly higher than the others values. Accordingly, the mean calculated without these two estimates was 6.6 Tg N yr^−1^ (10.4 Tg N_2_O yr^−1^; CV = 22%, *N* = 9). The base years of the estimates compiled in Figure 5 vary among the estimates. Moreover, it was found that the base year of each estimate is not always stated in each reference. Because the climate is changing, and interannual climate variation should not be regarded as being negligible, the difference in the selected base years should be an important consideration. In addition, the vegetation, land cover, or soil type that was masked out in each simulation varies among these studies, which is one of the sources of variations in the estimates. The compilation presented here provides approximate overall estimates based on historic reports; however, the consideration of the different calculation conditions used in various studies is one of the important process for lessening the variation of estimates among studies. Another issue is that the source of uncertainty and the definition of uncertainty differ among studies, which hinders the comparison of uncertainty in published estimates.

More distinct spatial distribution patterns and seasonality were found for CO_2_ and N_2_O than for CH_4_. This difference is mostly attributable to the high temperature sensitivity of CO_2_ and N_2_O and the low temperature sensitivity of CH_4_ in the model structure. Similar spatial distribution patterns were found in previous studies. For example, it is reported that the contribution of CO_2_ flux from tropical ecosystems was 67% [2]. Also it is estimated that more than 60% of the global N_2_O flux occurred via tropical forest and savanna ecosystems [6]. For the CH_4_ uptake flux, the global distribution pattern still appears to vary among models [4] [21]; some studies estimated distinct spatial distribution patterns, whereas others did not. For example, four schemes for CH_4_ uptake (the algorithms of *Potter et al.* (1996) [9], *Ridgewell et al.* (1999) [23], *Del Grosso et al.* (2000) [42], and *Curry* (2007) [22]) were used for global CH_4_ uptake flux estimates [21]; the comparison demonstrated that the total CH_4_ uptake fluxes estimated by the four schemes were comparable, but the fluxes showed the different spatial distribution patters.

One of the limitations of the model used in this study could be the simple exponential function that is used to estimate the temperature response of GHG fluxes, especially for the CO_2_ flux. It has been reported that the temperature sensitivity of soil CO_2_ fluxes changes depending on the temperature; in particular, it has been noted that soil CO_2_ flux shows a greater temperature sensitivity at low temperatures [43]. For simplicity, the gas flux submodels used here adopts a simple exponential temperature response. This simplification may lead to errors in the estimation of soil CO_2_ fluxes in cooler regions, although this limitation likely has a small effect on the global estimates because the contributions of temperate and tropical regions dominate the global soil CO_2_ flux. Another limitation is that our simulations did not distinguish between forested and agricultural areas. The gas flux submodels were parameterized using data observed in forested areas and do not include the effects of agricultural activity (e.g., N fertilizer sources). The N_2_O flux, in particular, substantially differs between forested areas and agricultural areas. Therefore, the estimates reported in this study only account for so-called background emissions from agricultural areas.

An advantage of the present study is that the estimates are based on the simple data-oriented models that were data-assimilated with multi-site data using Bayesian calibration; therefore, the model estimates were well constrained by the observed data and are shown with uncertainty. To obtain more data-constrained estimates of global soil GHG fluxes, however, it would be important to constrain models with the global dataset via the data-assimilation process. One of the key factors is the development of global datasets [44]. Another key is to include necessary, though not too many, processes in the model. Simpler models are easy to data-assimilate and can provide more data-constrained estimates, but they may not be good for long-term estimations because a variety of potential feedback processes should affect the fluxes. However, too many detailed-process-oriented models can provide possible feedback processes but are not easily data-constrained with global datasets, and they increase uncertainty. Therefore, to identify essential processes in describing global soil GHG fluxes, closer collaborations between modelers and experimenters/observers and inter-model comparisons are vital.

This study reported new global estimates of soil CO_2_ emission, CH_4_ uptake, and N_2_O emission fluxes, which were estimated using a simple data-oriented model. The estimates were comparable to the previous estimates for all of the gases evaluated. The simulation results clearly demonstrated differences and similarities in spatial distribution patterns and in the seasonality of the three gas fluxes. The results, including both previous and the present estimates, revealed that the reported estimates vary and have large uncertainties but that the overall magnitudes are in general agreement. To lessen the uncertainty in soil GHG flux estimates further, it is necessary to build global databases and identify key processes in describing global soil GHG fluxes.

## Materials and Methods

The SGR, a regional, simple soil greenhouse gas flux model, was used [8] [11]; the SGR model consists of submodels of soil temperature, water, and GHG fluxes (Figure S1). A monthly time step was adopted, and the inputs for the model were the monthly mean air temperature and the monthly precipitation. The soil physical and chemical properties were also required. The soil temperature submodel calculates the soil temperature using the mean air temperature and the snow cover, and the soil water submodel calculates the water-filled pore space (WFPS) using the air temperature, the potential evapotranspiration [45], and the precipitation. The soil water characteristic was estimated using the generalized soil-water relationship [46]. The Bayesian calibration scheme was used to parameterize the snow, soil temperature, WFPS, and soil gas submodels. The scheme is an optimization scheme that uses Monte Carlo sampling and a model-data synthesis scheme. In each grid, the snow cover and potential evapotranspiration were calculated using monthly air temperature and precipitation data, and the soil temperature and WFPS were subsequently simulated. Using the soil physiochemical property, WFPS, and soil temperature, the flux model for each gas yields a monthly flux. The model is described in detail elsewhere [8] [11]–[12], and all parameters are shown in Table S1.

### Gas Flux Submodel

The SG models were used for the soil GHG fluxes [11]. In these models, each gas flux (CO_2_, µg C m^−2^ s^−1^; CH_4_, µg C m^−2^ h^−1^; and N_2_O, µg N m^−2^ h^−1^) is described by the same three factors: soil physiochemical properties, soil water, and soil temperature:


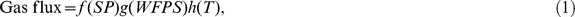


where *f*(*SP*) is the function for the soil physiochemical properties (*SP*, 0–5-cm soil layer), *g*(*WFPS*) is the function for the WFPS (5-cm depth), and *h*(*T*) is the function for the soil temperature (5-cm depth).

The *f*(*SP*) is defined as follows: the function for the CO_2_ flux was defined to increase with increasing C/N ratios (*CNR*, 0–5-cm soil layer):


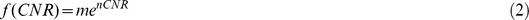


The function for the CH_4_ flux was defined to decrease with increasing bulk density (*BD*, Mg m^−3^, 0–5-cm soil layer):


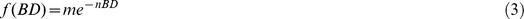


For the N_2_O flux, the function was defined to decrease with decreasing *CNR*:


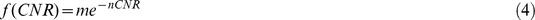


The function for the WFPS (5 cm) was defined by the following equation and was used for every gas model:


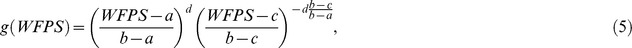


where the parameters *a* and *c* are the minimum and maximum values of the WFPS, respectively (i.e., *g*(*a*) = *g*(*c*) = 0). Parameter *b*, which ranges between *a* and *c*, is the optimum parameter (i.e., *g*(*b*) = 1). Parameter *d* controls the curvature of the function, but the three other parameters also affect the shape. The function has a convex shape, and the values range from 0 to 1.

The exponential function was used for the soil temperature for every gas flux as follows:


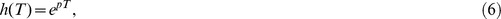


where *p* is the parameter and *T* is the soil temperature (°C, 5 cm). The value of *h*(*T*) is 1 when the soil temperature is 0°C.

The gas flux submodels were calibrated using multi-site data, which were gathered monthly in Japanese forests between 2002 and 2004 (36 sites, *N* = 768 in total for each gas flux) [11]. After parameterisation, the values of the root mean square errors (RMSE) for the CO_2_, CH_4_, and N_2_O fluxes were 10.25 µg C m^−2^ s^−1^, 29.29 µg C m^−2^ h^−1^, and 5.65 µg N m^−2 ^h^−1^ (*N* = 768 for each gas), respectively.

### Snow Submodel

I adopted a simple snow model that calculates the snow accumulation and snowmelt based on the air temperature and the precipitation [47].


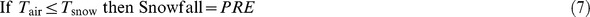



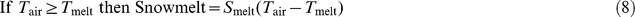


where *T*_air_ is the monthly air temperature (°C), *T*_snow_ is the maximum temperature at which precipitation becomes snow (°C), *T*_melt_ is the minimum temperature at which snowmelt occurs (°C), *S*_melt_ is the snow melting rate (mm°C^−1^), and *PRE* is the precipitation (mm). This simple snow model was used to estimate whether soil is covered with snow. In this model, the amount/depth of snow accumulation does not affect the simulation. Instead, the model output is affected by whether the soil is covered with snow via the soil temperature submodel.

### Soil Water Submodel

Because the gas flux models require the WFPS, the WFPS was calculated in the soil water submodel. First, an index of wetness was defined as follows:


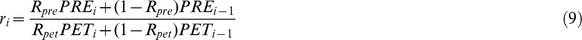


where *r*_i_ is the wetness index of the month (ratio). *PRE*_i_ and *PRE*_i−1_ are the precipitation for the month and the last month (mm), respectively, and *PET*_i_ and *PET*_i−1_ are the potential evapotranspiration of the month and the last month (mm), respectively. *R*_pre_ and *R*_pet_ are constants (ratio) that indicate the weights of the precipitation and potential evapotranspiration of the month, respectively. The function indicates that the wetness of the site, *r*_i_, is affected by not only the precipitation and potential evapotranspiration of the month but also those of the last month.

Second, the WFPS was calculated using the following functions:


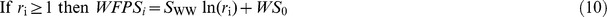



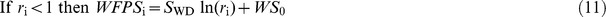



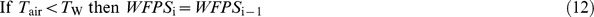


where *WS*_0_ is a WFPS when *r* is 1 (or ln(*r*) = 0) and is defined as the WFPS of a 30-kPa soil water potential and *WFPS*_i−1_ is the WFPS of the last month. It is assumed that the WFPS does not change when the air temperature is low (lower than *T*_W_ °C ) because of the low evapotranspiration and the minor amount of snowmelt.

The potential evapotranspiration was estimated using the Thornthwaite method [45], which calculates the potential evapotranspiration using the air temperature and the longitude. The generalized soil−water characteristics model [46] was used to calculate the soil water characteristics (*WS*_0_) from the soil texture. The default parameters were used for the potential evapotranspiration submodel [45] and the soil water characteristics submodel [46].

### Soil Temperature Submodel

A linear model was used for soil temperature (*T*_soil_, °C): when the soil is not covered with snow, the soil temperature is calculated with a linear function of air temperature (*T*_air_, °C); when soil is covered with snow, a constant temperature was assumed.


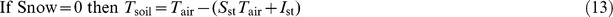



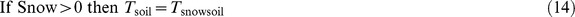


where *S*_st_, *I*_st_, and *T*_snowsoil_ are constant (°C).

### Effect of Atmospheric CH_4_ Concentration on CH_4_ Uptake

Although uncertain feedbacks between soil nitrogen and CH_4_ oxidation in soil have been suggested [48], the CH_4_ uptake is generally expected to increase with the atmospheric CH_4_ concentration [4]. The effect of atmospheric CH_4_ was therefore included by multiplying the factor of CH_4_ concentration, *j*([CH_4_]), which was calculated using the relative concentration of atmospheric CH_4_.


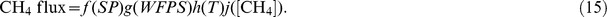


### Driving Data and Simulations

The gas fluxes were evaluated with a spatial resolution of 0.5°×0.5°. The air temperature and precipitation were derived from the CRU 3.1 (Climate Research Unit) climate data [49], and the global grid area data in the EOS-WEBSTER were used. The ISRIC-WISE global dataset of soil properties was used for the distribution of the soil physiochemical properties [50]. The soil physiochemical properties in the ISRIC-WISE dataset were converted to those of the 0–5-cm soil layer using ISRIC-WISE global soil profile data [51]. Soils with distinctively small bulk density (≤0.28 Mg m^−3^ in ISRIC-WISE) were excluded because they were presumed to be peat soils. The data of atmospheric CH_4_ concentrations observed at the Ryori BAPMon station, from the GLOBALVIEW-CH4 database [52], were used to calculate *j*([CH_4_]).

A Monte Carlo approach was used to evaluate the uncertainty of the estimates. For each simulation, new parameters were chosen from the uncertainty for each parameter, as determined through the Bayesian calibration. A normal distribution with a 10% coefficient of variance was assumed for each parameter that did not undergo Bayesian calibration. The model was run 1000 times, and the results were analyzed using the R statistical computing software (version 2.11.1). The codes for the SGR and Bayesian calibration were written in C.

Here, the average CO_2_ emission flux, CH_4_ uptake flux, and N_2_O emission flux between 1980 and 2009 are shown. The SGR models do not include CH_4_ emissions; therefore, this study focuses on the soil CH_4_ uptake. Areas of ice, permanent water, mangrove, and peat soils (see above) were masked out. The cultivated area was included in this study.

### Comparison with Data from a Global Database of Soil CO_2_ Flux (Soil Respiration)

A global database of soil CO_2_ flux (soil respiration) was released recently (https://code.google.com/p/srdb/) [44]. Although the mismatch in scale between site-scale measurements and the coarse resolution of the simulation (0.5°×0.5°) should be an issue, the results of the simulation were compared with the data in the database (version 20100517a). For the comparison, the data from non-agricultural ecosystems without experimental manipulation measured using infrared gas analyzer or gas chromatography were extracted. The data with quality check flags, except for Q01, Q02, and Q03, were excluded (please see the database). A total of 1464 data points met the above conditions, and 1246 data points where the measurement locations (latitude and longitude) corresponded to the simulated area were included. The comparison showed that the two agreed in their magnitude and were positively correlated (*R* = 0.43) (Figure S2). However there was some mismatches: the variation in the simulated values was less than that of observed data points. In particular, the simulation did not produce large fluxes (e.g. >1500 g C m^−2^ yr^−1^). This difference is partly due to the different scale in the field measurements and the simulation. The second difference is that the fluxes generated by the simulation were smaller than those of the database. This difference resulted in the gap between the estimate in this study and the global estimate reported by Bond-Lamberty (2010) [2], which is based on the global database (Figure 5). This gap would suggest that the global estimate substantially varies depending on the data used to constrain the model, although the differences in model structures and the scale mismatch between measurements and simulations should be taken into account.

## Supporting Information

Figure S1 Schematic diagram of the modeling approach.
(DOC)

Figure S2 Comparison between data in a global dataset [44] and those of the simulations. The data from non-agricultural ecosystems without experimental manipulation measured using infrared gas analyzer or gas chromatography were extracted. The data with quality check flags, except for Q01, Q02, and Q03, were excluded (please see the database). A total of 1464 data points met the above conditions, and 1246 data points where the measurement locations (latitude and longitude) corresponded to the simulated area were included. The broken line is y = 0.17x+418 (*P*<0.0001). The Pearson’s correlation coefficient was 0.43.
(DOC)

Table S1 Definitions and values of the parameters in the model [8] [11].
(DOC)
