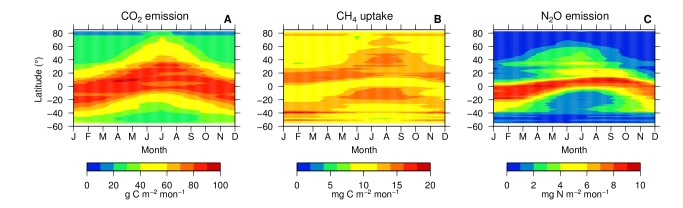# Correction: A New Estimation of Global Soil Greenhouse Gas Fluxes Using a Simple Data-Oriented Model

**DOI:** 10.1371/annotation/4526d447-db8e-4bb7-831c-752b8498a63d

**Published:** 2012-11-06

**Authors:** Shoji Hashimoto

There are errors in Figure 4. The correct figure can be found here: 

**Figure:**