# Supplementary figures and images for: A New Estimation of Global Soil Greenhouse Gas Fluxes Using a Simple Data-Oriented Model
Source: PLoS One. 2012 Aug 2;7(8):e41962. doi: 10.1371/journal.pone.0041962 (PMC3410890; doi:10.1371/journal.pone.0041962)

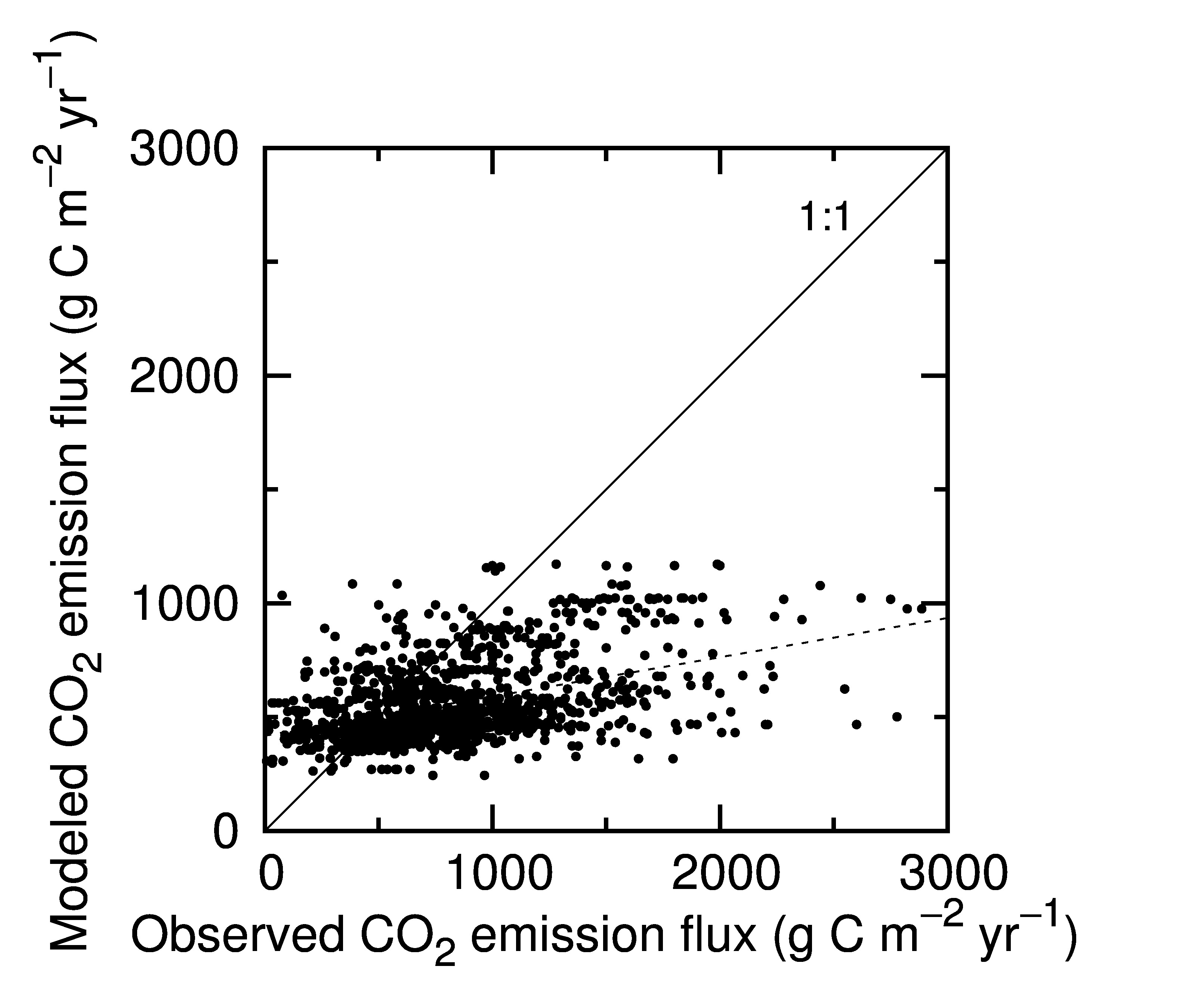

Supplement: Figure S2 — Comparison between data in a global dataset [44] and those of the simulations. The data from non-agricultural ecosystems without experimental manipulation measured using infrared gas analyzer or gas chromatography were extracted. The data with quality check flags, except for Q01, Q02, and Q03, were excluded (please see the database). A total of 1464 data points met the above conditions, and 1246 data points where the measurement locations (latitude and longitude) corresponded to the simulated area were included. The broken line is y = 0.17x+418 (P<0.0001). The Pearson’s correlation coefficient was 0.43. (DOC) [file pone.0041962.s002.doc]
