# Supplementary material for: A New Estimation of Global Soil Greenhouse Gas Fluxes Using a Simple Data-Oriented Model
Source: PLoS One. 2012 Aug 2;7(8):e41962. doi: 10.1371/journal.pone.0041962 (PMC3410890; doi:10.1371/journal.pone.0041962)
Supplement: Table S1 — Definitions and values of the parameters in the model [8] [11]. (DOC) [file pone.0041962.s003.doc]

| Parameter (Symbol) | Definition | Unit | Value |
| --- | --- | --- | --- |
|  | Snow submodel |  |  |
| *T*_snow_ | Maximum temperature at which precipitation becomes snow | °C | 3.0 |
| *T*_melt_ | Minimum temperature at which snow melt occurs | °C | −4.3 |
| *S*_melt_ | Snow melting rate | mm °C^−1^ | 18.0 |
|  | WFPS submodel |  |  |
| *R*_pre_ | Ratio of the precipitation of the month | - | 0.62 |
| *R*_pet_ | Ratio of the PET of the month | - | 0.01 |
| *S*_WW_ | Slope of the relationship between the wetness index and WFPS (*r* ≥1) | - | 0.044 |
| *S*_WD_ | Slop of the relationship between the wetness index and WFPS (r<1) | - | 0.143 |
| *Min*_pet_ | Minimum PET | mm | 2.95 |
| *T*_w_ | Temperature below which WFPS does not change | °C | −0.458 |
|  | Soil temperature submodel |  |  |
| *S*_st_ | Slope of the relationship between air and soil temperature | - | 0.19 |
| *I*_st_ | Intercept of the relationship between air and soil temperature | °C | −1.6 |
| *T*_snowsoil_ | Soil temperature when soil is covered by snow | °C | 1.7 |
|  | Gas submodel (CO_2_) |  |  |
| *m* | Constant for the relationship between C/N ratio and flux | μg C m^−2^ s^−1^ | 4.95 |
| *n* | Constant for the relationship between C/N ratio and flux | - | 0.033 |
| *a* | Minimum WFPS | - | −0.10 |
| *b* | Optimum WFPS | - | 0.50 |
| *c* | Maximum WFPS | - | 2.69 |
| *d* | Shape parameter for WFPS function | - | 1.52 |
| *p* | Temperature sensitivity | - | 0.067 |
|  | Gas submodel (CH_4_) |  |  |
| *m* | Constant for the relationship between bulk density and flux | μg C m^−2^ h^−1^ | 223.2 |
| *n* | Constant for the relationship between bulk density and flux | - | 1.8 |
| *a* | Minimum WFPS | - | −2.0 |
| *b* | Optimum WFPS | - | 0.07 |
| *c* | Maximum WFPS | - | 1.1 |
| *d* | Shape parameter for WFPS function | - | 4.3 |
| *p* | Temperature sensitivity |  | 0.009 |
|  | Gas submodel (N_2_O) |  |  |
| *m* | Constant for the relationship between C/N ratio and flux | μg N m^−2^ h^−1^ | 48.6 |
| *n* | Constant for the relationship between C/N ratio and flux | - | 0.25 |
| *a* | Minimum WFPS | - | −0.41 |
| *b* | Optimum WFPS | - | 1.0 |
| *c* | Maximum WFPS | - | 1.8 |
| *d* | Shape parameter for WFPS function | - | 9.0 |
| *p* | Temperature sensitivity | - | 0.12 |
