# Supplementary material for: A New Estimation of Global Soil Greenhouse Gas Fluxes Using a Simple Data-Oriented Model
Source: PLoS One. 2012 Aug 2;7(8):e41962. doi: 10.1371/journal.pone.0041962 (PMC3410890; doi:10.1371/journal.pone.0041962)
Supplement: Supplementary file 3 [file pone.0041962.s001.doc]

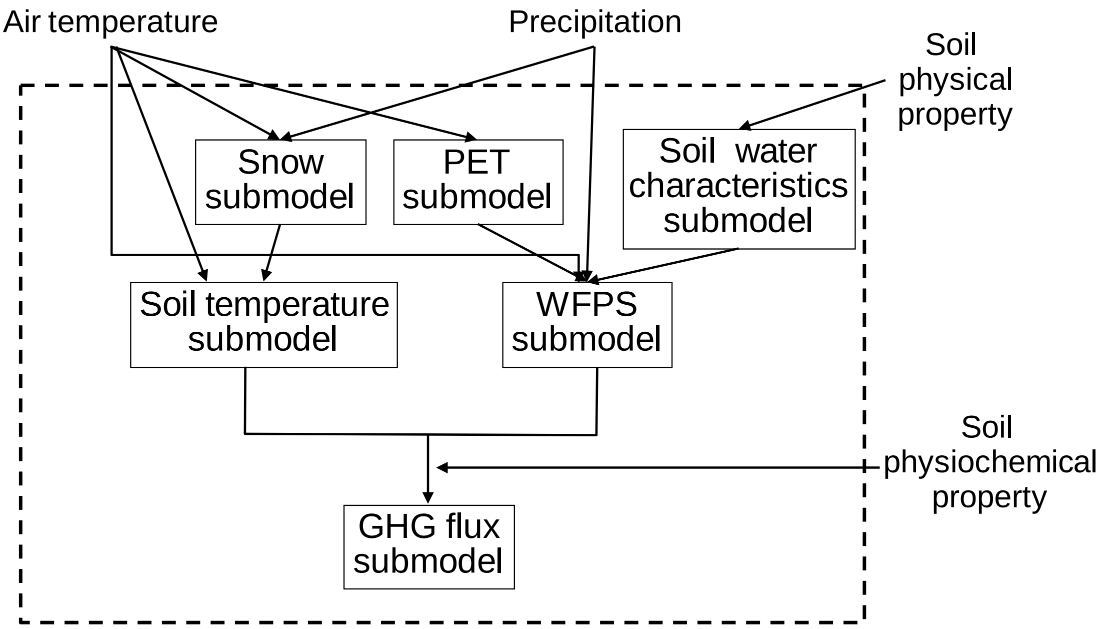

Air temperature
Precipitation
Soil physical property
Snow submodel
PET submodel
Soil water characteristics submodel
Soil temperature submodel
WFPS submodel
Soil physiochemical property
GHG flux submodel
